# Biocontrol potential of *Burkholderia* spp. against *Fusarium oxysporum* f. sp. *lycopersici* infecting tomato

**DOI:** 10.1186/s40529-026-00497-z

**Published:** 2026-08-28

**Authors:** Sunil Khayamali, Tao-Ho Chang, Kusum Mushyakhwo, Jenn-Wen Huang, Pi-Fang Linda Chang

**Affiliations:** 1 International Doctoral Program in Agriculture, College of Agriculture and Natural Resources, National Chung Hsing University, Taichung, 40227 Taiwan; 2 Program in Plant Health Care, Academy of Circular Economy, National Chung Hsing University, Nantou, 540216 Taiwan; 3 Department of Plant Pathology, College of Agriculture and Natural Resources, National Chung Hsing University, Taichung, 402202 Taiwan

**Keywords:** Beneficial microbes, Fusarium wilt, Silicate-solubilizing bacteria (SSB), *Solanum lycopersicum* L.

## Abstract

**Background:**

Fusarium wilt of tomato caused by *Fusarium oxysporum* f. sp. *lycopersici* (FOL) is a serious disease. Host resistance and soil fumigation may reduce the impact of FOL, but the effectiveness may decrease over time due to the diversity and evolution of pathogens. Silicon is the second most abundant element in the Earth’s crust, but has been overlooked in crop protection due to its poor availability to plants. Silicate-solubilizing bacteria (SSB) are reported to enhance soil nutrition, plant growth, and stress tolerance by transforming inorganic silicon into a plant-available form. Hence, SSB with antimicrobial properties can act as green alternatives to fungicides. Therefore, this study aims to isolate rhizospheric SSB and uncover their benefits in crop protection.

**Results:**

Among thirteen potentially beneficial strains identified through 16S rDNA sequencing, *Burkholderia contaminans* NRBC (NRBC) and *Burkholderia* sp. NRBA (NRBA) were chosen for greenhouse experiments to evaluate their effectiveness in promoting plant growth and suppressing FOL (race 2). Under normal greenhouse conditions, the co-application of NRBC and silicon significantly increased shoot dry weight. Under FOL stress, all bacteria-treated groups were taller than the control groups. Bacterial treatments significantly reduced disease incidence and suppressed disease progression with no significant effect of the silicon supplement. The expression profiling of some defense-related genes revealed that soil-inoculation with NRBA and NRBC induced transcriptional patterns consistent with the involvement of salicylic acid (SA) and jasmonic acid/ethylene (JA/ET)-related defense responses.

**Conclusions:**

SSB isolates, NRBA and NRBC, are effective standalone biocontrol agents for managing Fusarium wilt in tomato. Silicon supplementation did not show the anticipated plant growth promotion of tomato plants and FOL suppression.

**Supplementary Information:**

The online version contains supplementary material available at https://doi.org/10.1186/s40529-026-00497-z.

## Introduction

Tomato (*Solanum lycopersicum* L.) is a highly nutritious and economically important Solanaceae crop, which has been cultivated all over the world for direct consumption and agricultural commodities (Collins et al. 2022; Pazarlar et al. 2022). However, tomato production is compromised by several biotic and abiotic stresses (Du et al. 2022). Specifically, tomato Fusarium wilt, caused by *Fusarium oxysporum* f. sp. *lycopersici* (FOL), is one of the most destructive diseases affecting tomato production worldwide, resulting in approximately 80% yield losses (Hernandez-Aparicio et al. 2021; Nabi et al. 2021). Tomato producers rely heavily on fungicides to manage FOL, which increases production costs, environmental pollution, and associated health risks (Okungbowa and Shittu 2012; Du et al. 2022). Most of the conventional fungicides do not reach the xylem vessels, where FOL causes blockage (Pazarlar et al. 2022). Moreover, chlamydospores of FOL remain viable in harsh environments for several years (Gordon 2017). Cultural control methods, such as crop rotation and soil solarization, can reduce the pathogen density in the soil, but cannot eradicate the spores (Ajilogba and Babalola 2013). Breeding of wilt-resistant cultivars provides efficient control; however, the resistance might be weakened due to the diversity and dynamic co-evolution of pathogens (Akram et al. 2021; Hernandez-Aparicio et al. 2021; Du et al. 2022). Hence, finding an effective, economical, and eco-friendly solution is crucial. Beneficial microbes can act as biocontrol agents (BCAs) to control phytopathogens. BCAs exhibit versatile modes of action against FOL, such as competition for nutrients, direct parasitism, antibiosis, and induced plant resistance (Elanchezhiyan et al. 2018; Bhattacharya et al. 2019; Medeiros and Bettiol 2021; Zhang et al. 2021). In this study, we aimed to isolate and characterize silicate-solubilizing bacteria (SSB) with multiple plant growth-promoting traits. SSB transforms the inorganic form of silicates to the only plant-available form of silicon known as monosilicic acid (H_4_SiO_4_) (Chandrakala et al. 2019). Soil Si amendment has potential benefits in crop protection, but such practice has been overlooked due to its inorganic nature in the soil (Wang et al. 2017; Adhikari et al. 2020). Therefore, identification and application of SSB with antifungal properties not only enhance disease tolerance but also replenish soil nutrients utilizing natural sources.

This approach could maximize the utilization of silicon (Si), which is the second-most abundant element on the Earth, making up 28% of the Earth’s crust. Silicon accumulators, such as rice, benefit from the active transport and deposition of monosilicic acid (H_4_SiO_4_) in the plant tissues, forming a physical barrier to prevent pathogen invasion (Ma et al. 2006). Tomato plants are poor Si accumulators (Mitani and Ma 2005) due to a lack of functional Si efflux transporter Lsi2 in the root (Sun et al. 2020). Nevertheless, Si supplementation has been reported to increase the stress tolerance in tomato plants by indirect mechanisms (Shivaraj et al. 2022). Applying Si-rich rice husk to soil enhances tomato resistance to anthracnose by increasing cuticle thickness and fruit firmness (Somapala et al. 2016). Si application increases salicylic acid (SA) and citric acid accumulation in response to bacterial wilt infection in tomato plants, thereby suppressing pathogens (Fan et al. 2018). Si treatment significantly increases Si contents in tomato roots and reduces the incidence of crown rot and root rot caused by *F. oxysporum* f. sp. *radicis-lycopersici* in tomato plants (Huang et al. 2011). Hence, tomatoes, a typical Si non-accumulating species, can also benefit from Si supplementation and accumulation in the root.

Given the independent roles of SSB and inorganic Si in promoting plant defense, this research is aimed to evaluate their co-application as a potential strategy for managing FOL in tomato. Therefore, two best-performing *Burkholderia* spp. among isolated SSB were selected and co-applied with Si to evaluate their potential to biocontrol Fusarium wilt infection in tomato plants in the greenhouse, where both strains suppressed the disease. The defense-related gene expression analysis revealed altered plant hormonal pathways due to the interaction of SSB and Si.

## Materials and methods

### Isolation of rhizobacteria

Rhizobacteria were isolated from the soil samples collected from four organic fields in Pingtung County, Taiwan (Fig. S1), following the method described by Lee et al. (2019). Briefly, one gram of debris-free soil was mixed in 9 mL of sterile distilled water (dH_2_O), homogenized at 100 rpm for 30 min, and diluted fivefold. Then, 100 µL of each dilution was spread on nutrient agar (NA) medium plates containing 3 g/L beef extract, 5 g/L peptone, and 2% agar, and the plates were incubated at 30 ± 2 °C for 3 days in the dark. Morphologically different colonies were independently picked, purified on NA, and preserved in 25% glycerol stock at − 80 °C (Gao et al. 2017) for subsequent use.

### In vitro screening of rhizobacteria for plant growth-promoting properties

Bacterial strains were cultured on NA medium at 30 ± 2 °C for 2 days. Unless otherwise stated, the bacterial cells were suspended in sterile distilled water (dH_2_O), and the concentration was adjusted to 10^7^ colony-forming units (CFU)/mL.

#### Silicate and phosphate-solubilizing index

**Fig. 1:**
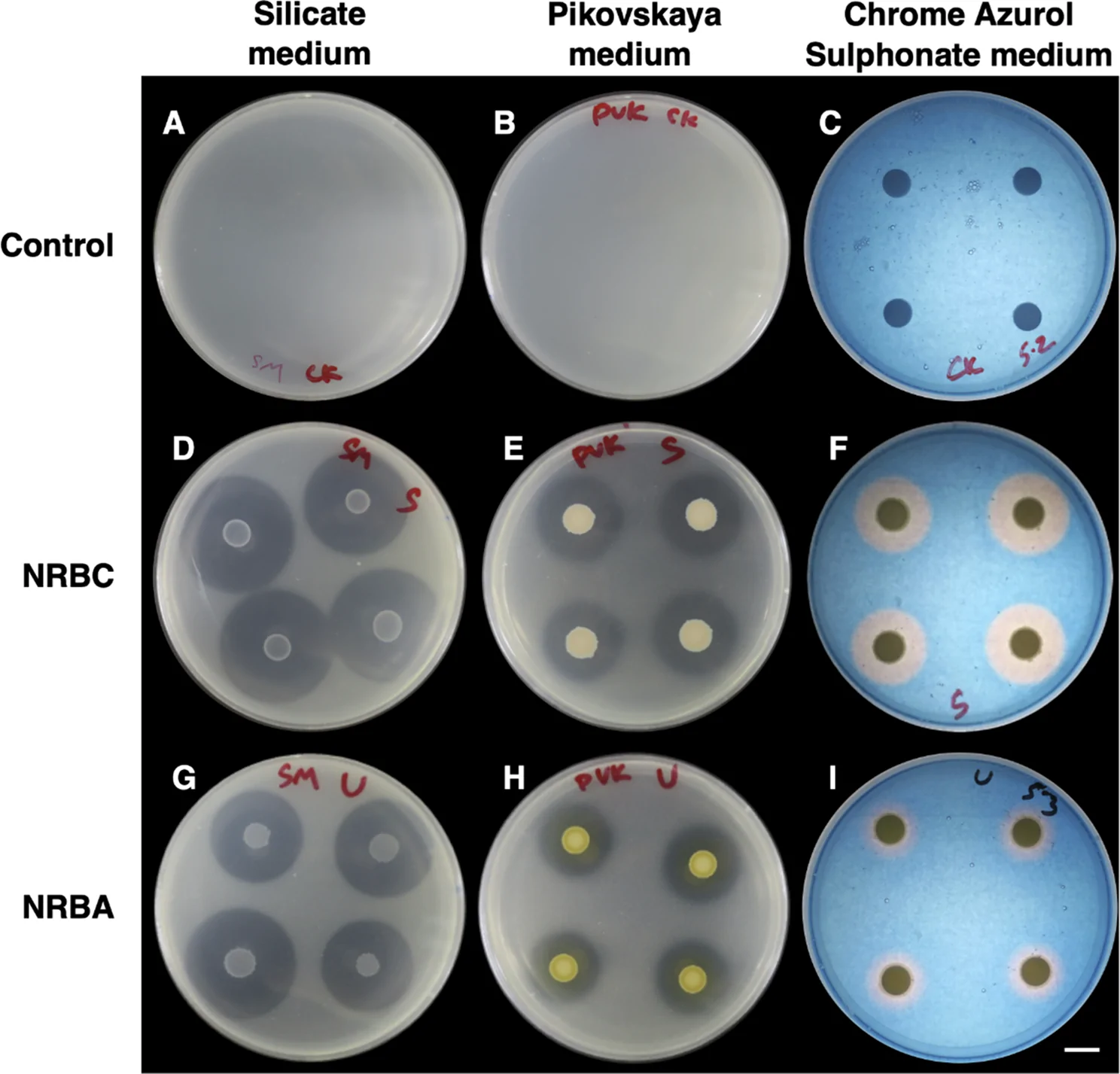
In vitro beneficial traits exhibited by *Burkholderia contaminans* NRBC and *Burkholderia* sp. NRBA. Five microliters of bacterial suspensions (10^7^ CFU/mL) or sterile distilled water were added at four spots on a silicate medium (**A**,** D**,** G**), Pikovskaya medium (**B**,** E**,** H**), and a 10-mm sterile filter paper disc placed on the Chrome Azurol Sulphonate (CAS) agar medium (**C**,** F**,** I**). No halo zone was observed in the uninoculated control (**A**,** B**, and **C**). The appearance of a halo-zone around the bacterial colony indicates (**D**, **G**) silicate solubilization, (**E**,** H**) phosphate solubilization, and (**F**,** I**) siderophore production. A scale bar at the bottom right corner represents 1 cm

The silicate-solubilizing ability of the strains was determined by spot-inoculating five microliters of bacterial suspension on a silicate medium (SM) containing 0.25% magnesium trisilicate (Mg_2_O_8_Si_3_) (Kang et al. 2017). Plates were incubated at 30 ± 2 °C for 7 days, and the diameter of transparent halos around the colonies (Fig. 1D and G) was measured. The strains exhibiting silicate solubilization were further examined for their phosphate-solubilizing ability on Pikovskaya (PVK) medium (HiMedia, Mumbai, India) containing 0.5% tricalcium phosphate [Ca_3_(PO_4_)_2_] (Ouattara et al. 2019). Plates were incubated at 30 ± 2 °C for 7 days, and the diameter of transparent halos around the colonies (Fig. 1E and H) was measured. Silicate-solubilizing index (SSI) (Santi and Goenadi 2017) and phosphate-solubilizing index (PSI) (Li et al. 2023) were determined as the diameter of the halo-zone (mm)/diameter of the colony (mm) (Akintokun et al. 2007; Cruz et al. 2022).

Two candidate bacterial strains were evaluated for their ability to solubilize inorganic silicates and release soluble silicon in a broth assay, as described by Chandrakala et al. (2019) with modifications. Diatomaceous earth (SiO_2_) and field soil were used as insoluble silicate sources. The field soil was ground into powder and passed through a 2-mm sieve before use. The basal medium (per liter) consisted of 20 g glucose, 1 g ammonium sulfate, 0.2 g potassium dihydrogen phosphate, and 0.1 g magnesium sulfate, and was supplemented with 0.25% (w/v) of each insoluble silicate source. The pH was adjusted to 7 before sterilization. Each bacterial strain was inoculated into 10 mL of medium in a 50 mL Falcon tube to a final concentration of 10^7^ CFU/mL and incubated in the dark at 28 °C. After 7 days of incubation, cultures were centrifuged at 10,000 × *g* for 10 min, and the supernatants were collected for the determination of soluble silicon content using the molybdate blue colorimetric method (Kraska and Breitenbeck 2010).

#### Siderophore production

The ability of bacterial strains to produce siderophores was evaluated on the Chrome Azurol Sulphonate (CAS) agar medium as described by Ambrosini and Passaglia (2017). Five microliters of bacterial suspension were added to a 10-mm sterile filter paper disc placed on the CAS agar medium, and incubated at 30 ± 2 °C for 3 days in the dark. The color change of the CAS medium from blue to orange around the colony was considered siderophore production (Fig. 1F and I). The siderophore production index (SPI) was calculated as the diameter of the halo zone (mm)/colony diameter (mm).

#### Indole-3-acetic acid (IAA) production

The ability of bacterial strains to produce IAA was assessed as described by Gang et al. (2019) with modifications. Briefly, 100 µL of bacterial culture (10^8^ CFU/mL) was inoculated into 3 mL nutrient broth (NB) amended with or without 0.15% (w/v) L-tryptophan, and incubated at 30 °C in the dark for 2 days with continuous oscillation at 120 rpm. After centrifugation at 8000 × *g* for 10 min, 1 mL supernatant was mixed with an equal volume of Salkowski’s reagent and incubated at room temperature for 30 min in the dark. The development of pink color in culture filtrate amended with L-tryptophan confirmed the IAA production. To measure IAA concentration, the color intensity (OD_536_) was measured using a microplate spectrophotometer (BioTek Epoch 2, Vermont, USA) and compared with the standard curve obtained from known concentrations of IAA (0 to 60 µg/mL).

#### Antifungal property

The antifungal properties of bacterial strains against five major phytopathogenic fungi (Table S1) were determined using a dual culture technique based on Fouda et al. (2021) with revisions suggested by Ouattara et al. (2019). Briefly, five-microliter bacterial suspensions were streaked on three equidistant sides of potato dextrose agar (PDA) (Difco, Sparks, Maryland, USA). PDA plates were streaked with dH_2_O as a control. After incubating the agar plates at 28 ± 2 °C for 24 h, a 5-mm fungal disk from actively growing edge of a 10-day-old fungus was placed at the center, and incubated until the fungal mycelium grew all over the medium on the control plates. The relative fungal growth inhibition (%) was calculated using the equation ((Rc-R)/Rc) ×100, where “Rc” is the radius of the fungus on the control plate, and “R” is the radius of the fungus on the bacterium-inoculated plate.

### Molecular identification of bacterial strains

#### 16S rRNA gene sequencing

For the preliminary identification of 18 isolated strains (Table S2), near full-length 16S rRNA gene sequences were amplified using the universal primer set 16SF forward primer (5′-CCGAATTCGTCGACAACAGAGTTTGATCCTGGCTCAG-3′) and 16SR reverse primer (5′-CCCGGGATCCAAGCTTACGGCTACCTTGTTACGACTT-3′). The 50 µL reaction contained 5 µL 10X *Taq* reaction buffer (with MgCl_2_) (Cyrusbioscience, Taipei, Taiwan), 2 µL each of 10 µM forward and reverse primer, 0.5 µL of *Taq* DNA polymerase (Cyrusbioscience, Taipei, Taiwan), and 39.5 µL of ddH_2_O. The PCR amplification was conducted using SensoQuest Labcycler, Göttingen, Germany with an initial denaturation at 94 °C for 5 min, followed by 30 cycles of denaturation at 94 °C for 1 min, annealing at 53 °C for 45 s, extension at 68 °C for 90 s, and a final extension at 68 °C for 10 min (Chandrakala et al. 2019). The bacterial colony was used as a template (Hwang et al. 2021). The PCR products were electrophoresed in a 1.5% agarose gel alongside a 100 bp ladder (BioKit, Miaoli, Taiwan) in 0.5X TAE buffer (BioMi, Taichung, Taiwan) at 100 Volts for 30 min, stained with 0.5 µg/mL ethidium bromide (EtBr) (AMRESCO, Solon, Ohio, USA), and viewed under the UV light. The PCR products were sequenced by Mission Biotech (Taipei, Taiwan). The raw sequences were quality-trimmed and reassembled using BioEdit v 7.2.5 software (Cruz et al. 2022). The assembled sequences were subjected to a homology test with the bacteria-specific genes to obtain the closest identity using the BLAST (Basic Local Alignment Search Tool) program of the NCBI database available at https://blast.ncbi.nlm.nih.gov/Blast.cgi.

#### Multilocus sequence analysis (MLSA)

Two bacterial strains, NRBC and NRBA, that exhibited multiple beneficial in vitro traits and strong antagonistic activity against all tested phytopathogens, including *F. oxysporum* f. sp. *lycopersici*, were selected for further study. Based on 16S rRNA gene sequence analysis (Table S2), both isolates were classified within the *Burkholderia cepacia* complex (Bcc). Since 16S rRNA gene sequences generally lack sufficient resolution for species-level differentiation within the *Burkholderia cepacia* complex, the strains were further characterized by multilocus sequence analysis (MLSA) using seven housekeeping genes, as described by Spilker et al. (2009). A phylogenetic tree was constructed in MEGA11.0 (Tamura et al. 2021) based on 2760 bp concatenated sequence comprising fragments of seven housekeeping genes: *atpD* (440 bp), *gltB* (400 bp), *gyrB* (450 bp), *lepA* (396 bp), *phaC* (384 bp), *recA* (393 bp), and *trpB* (297 bp). The housekeeping gene sequences of representative type and reference strains were obtained from Bcc PubMLST database (https://pubmlst.org/bcc/) and NCBI GenBank.

### Greenhouse experiments

#### Preparation of tomato plants and bacterial suspensions

Tomato seeds (cv. Farmers 301; Known-You Seed Co., Ltd., Kaohsiung, Taiwan), susceptible to FOL (race 2) (Li et al. 2018), were surface-sterilized by soaking in 3% sodium hypochlorite for 15 min and rinsed 10 times with sterile dH_2_O (Hernandez-Aparicio et al. 2021). The sterilized seeds were pre-germinated on a moistened filter paper in Petri dishes under darkness for 2 days. The sprouted seeds were sown in seed plugs (4 cm × 4 cm) filled with peat moss (BVB substrates, Coldenhovelaan, The Netherlands) and allowed to germinate in a growth chamber at 28 °C with a 16/8 h photoperiod for 10 days.

Bacteria were mass cultured in Tryptic Soy Broth (TSB) (Difco, Sparks, Maryland, USA) at 30 °C with continuous oscillation at 180 rpm in the dark for 2 days, harvested by centrifugation at 8000 × *g* for 10 min, and resuspended in sterile dH_2_O to obtain 10^8^ CFU/mL.

#### Evaluation of beneficial microbes under greenhouse conditions

Ten-day-old tomato seedlings were individually transplanted into 10 cm plastic pots filled with a 100 g mixture of peat moss, vermiculite, and perlite (6:3:1). On the same day, half of the seedlings were supplemented with 20 mL/pot of 2 mM Si as potassium silicate (K_2_SiO_3_; AUO Crystal Corporation, Taichung, Taiwan). The other half, assigned as the non-silicon group, received potassium chloride (KCl) to balance potassium. Additionally, all plants were fertigated with 20 mL/pot of NPK fertilizer (HYPONeX 5, Taipei, Taiwan) diluted at the rate of 1 g/L. The pH of nutrient supplements was maintained within the range of 6.5 to 7.5 before application.

Two days after transplanting (DAT), both groups were soil-drenched with either 10 mL of bacterial suspension or dH_2_O per pot. The bacterial suspension containing 20-fold diluted cell-free culture broth (Chai et al. 2022) was reapplied at 9 DAT. Inoculation with FOL (Foly4 isolate) was performed the following day. The 10-day-old Foly4 isolate grown on half-strength sugar PDA at 28 °C under complete darkness was flooded with 10 mL of 0.05% Tween20, scrapped with a sterile spatula, and the suspension was filtered through a double layer of Mira cloth. The concentration of conidia in the obtained suspension was determined using a hemocytometer (Improved Neubauer, Lauda-Königshofen, Germany) and diluted with 0.05% Tween20 to achieve the spore concentration of 10^6^ spores/mL. The roots of each plant were lightly wounded, and half of the plants in each group were soil-drenched with 10 mL per pot of FOL spore suspension with 0.05% Tween 20, while the remaining half were mock-inoculated with 0.05% Tween 20. This setup resulted in a total of 12 treatment groups. Each group consisted of at least five replicate plants arranged in a completely randomized design (CRD) in the greenhouse. Tomato plants were grown at an average temperature of 28 ± 5 °C and relative humidity (RH) of 80 ± 5%, under fluorescent light providing a 16/8 h (light/dark) photoperiod to supplement natural sunlight.

The plant parameters were recorded at 21 dppi (days post-pathogen inoculation). The disease incidence, calculated as (number of infected plants/total number of plants) × 100%, was recorded at 14 and 21 dppi. Disease severity was rated at 8, 11, 14, 17, and 21 dppi by assessing the proportion of wilted or symptomatic leaves using a 0 to 4 scale (Shanmugam and Kanoujia 2011), where 0 = no symptoms; 1 = 0–25% of leaves with symptoms or leaf epinasty; 2 = 25–50% of leaves with symptoms; 3 = 50–75% of leaves with symptoms; and 4 = 75–100% of leaves with symptoms or plant death. The disease severity index was calculated using the following formula:

$$\begin{gathered}{{\mathrm{Disease\,severity\,index}}}\ (\% ) \hfill \\= \frac{{{\text{}}\sum \left( {Rating\, \times \,number\,of\,plant\,rated} \right)}}{{Total\,number\,of\,plants\, \times \,highest\,rating}}\, \times \,100 \hfill \\ \end{gathered} $$

The area under the disease progress curve (AUDPC) was estimated using the trapezoidal integration method (Campbell and Madden 1990).

#### *Fusarium oxysporum* f. sp. *lycopersici* (FOL) recovery assay

After disease scoring, hypocotyl tissues near the shoot base were collected for FOL recovery as previously described, with minor modifications (Jung et al. 2013; Constantin et al. 2019). Briefly, approximately 1 cm hypocotyl segments were surface-sterilized with 70% ethanol for 30 s, followed by 3% sodium hypochlorite for 90 s, and rinsed 10 times with sterile dH_2_O. The outer ends of each segment were trimmed, and the remaining tissue was aseptically transferred onto peptone-pentachloronitrobenzene (PCNB) medium, a selective medium for *Fusarium* spp. (Jung et al. 2013). Plates were observed for fungal growth 10 days after incubation at 28 °C in the dark.

### Quantitative reverse-transcription PCR (qRT-PCR) analysis of defense-related gene expression

Differential expression patterns of six defense-related genes (Table S3) were analyzed to identify the key molecular mechanisms underlying tomato Fusarium wilt suppression. Total RNA was extracted from hypocotyl tissues (three biological replicates), collected at 0, 4, and 8 dppi, using the FavorPrep™ plant total RNA mini kit (Favorgen Biotech Corp., Pingtung, Taiwan), following the manufacturer’s instructions. Three hypocotyls from different plants were pooled for RNA extraction. First-strand cDNA was synthesized from 1 µg total RNA using PrimeScript™ RT reagent Kit (Perfect Real Time) (TaKaRa, Kusatsu, Japan). Quantitative PCR was performed using a 15 µL reaction mixture containing 7.5 µL KAPA SYBR^®^ FAST qPCR Master Mix (2X) (KAPABIOSYSTEMS, Cape Town, South Africa), 1 µL cDNA, and 1 µL each of 10 mM forward and reverse primers on a Rotor-Gene Q (QIAGENE, Maryland, USA) real-time PCR cycler. The qRT-PCR condition was 95 °C for 5 min, followed by 40 cycles of 95 °C for 10 s, and 55 °C for 20 s. The *β*-tubulin gene (*TUB*) served as an internal control to normalize the expression level. Relative expression levels were calculated using the 2^−ΔΔCt^ method (Livak and Schmittgen 2001).

### Statistical analysis

Statistical analyses were performed with R version 4.1.2 (R Core Team 2021; Posit team 2023). Results are presented as means ± standard error of the mean (SEM) obtained from at least three independent experiments containing at least three replicas per trial. Each dataset was subjected to a normality and homogeneity of variance test using the Shapiro-Wilk test and Levene’s test, respectively. The parametric datasets were examined for statistical differences using one-way ANOVA, followed by either Duncan’s new multiple range test or Tukey’s honestly significant difference at *p* < 0.05, whereas the non-parametric data were analyzed using the Kruskal-Wallis test, followed by multiple pairwise comparisons with Dunn’s *post hoc* test at a significance level of *p* < 0.05. Benjamini-Hochberg (BH) method was used to adjust the *p*-values for multiple pairwise comparisons.

## Results

### Isolation, selection, and identification of rhizobacteria

A total of 68 rhizobacteria with plant growth-promoting abilities were isolated from the soil samples. The strains were categorized based on their morphology, SSI, PSI, and antifungal properties to select superior strains for sequencing. The 16S rDNA gene sequencing of 18 strains revealed that the isolated strains had above 99% identity with the type strains of six bacterial genera, namely, *Acinetobacter*, *Burkholderia*, *Klebsiella*, *Pseudomonas*, *Ralstonia* and *Serratia* (Table S2). *Pseudomonas* was the most dominant genus, which was present in soil samples from all locations except the NPUST rice field. Since some strains had a common identity with the type strains, only the best-performing strain was chosen for further experiments. Therefore, only 13 strains (Table 1) were selected for further analyses.

**Table 1:** In vitro beneficial properties of thirteen unique rhizobacterial strains

Bacterial strains	Nutrient solubilization traits			Plant growth-promoting traits	
	Silicate- solubilizing index (SSI) ^I^	Classification of SSI ^II^	Phosphate- solubilizing index (PSI) ^III^	Siderophore production index (SPI) ^IV^	Indole acetic acid production (µg/mL)
*Pseudomonas taiwanensis* WRPT	2.17 ± 0.09 ^e^	intermediate	1.85 ± 0.13 ^cd^	1.24 ± 0.03 ^c^	26.71 ± 2.51 ^cd^
*Serratia surfactantfaciens* WRSS	2.03 ± 0.11 ^e^	intermediate	1.77 ± 0.04 ^d^	-	6.50 ± 0.78 ^j^
*P. nitritireducens* WRPN-1	2.47 ± 0.12 ^de^	intermediate	2.30 ± 0.06 ^bc^	1.27 ± 0.03 ^c^	30.82 ± 0.53 ^bc^
*Acinetobacter baumannii* WRAB	4.10 ± 0.10 ^a^	high	3.00 ± 0.10 ^a^	-	19.92 ± 1.02 ^ef^
*P. mosselii* WRPM	2.38 ± 0.10 ^de^	intermediate	1.45 ± 0.03 ^d^	1.25 ± 0.04 ^c^	48.72 ± 1.99 ^a^
*P. nitroreducens* WSPN-21	2.09 ± 0.07 ^e^	intermediate	2.53 ± 0.16 ^ab^	1.44 ± 0.02 ^b^	32.11 ± 2.84 ^b^
*A. pittii* NRAP-1	3.13 ± 0.03 ^bc^	intermediate	2.33 ± 0.23 ^bc^	-	12.14 ± 0.30 ^hi^
*Burkholderia contaminans* NRBC	3.66 ± 0.20 ^ab^	intermediate	2.96 ± 0.40 ^a^	2.23 ± 0.02 ^a^	8.10 ± 0.62 ^ij^
*Burkholderia* sp. NRBA	3.31 ± 0.31 ^bc^	intermediate	2.34 ± 0.14 ^bc^	1.52 ± 0.04 ^b^	24.41 ± 3.51 ^de^
*Klebsiella variicola* NRKV	2.91 ± 0.21 ^cd^	intermediate	1.72 ± 0.06 ^d^	-	18.54 ± 2.10 ^fg^
*Ralstonia syzygii* subsp. *indonesiensis* NRRS	2.81 ± 0.37 ^cd^	intermediate	1.37 ± 0.10 ^d^	-	11.76 ± 0.40 ^hi^
*Acinetobacter pittii* LCAP	3.26 ± 0.24 ^bc^	intermediate	2.58 ± 0.05 ^ab^	-	14.22 ± 0.20 ^gh^
*Pseudomonas aeruginosa* LCPA	3.23 ± 0.10 ^bc^	intermediate	2.45 ± 0.13 ^b^	2.22 ± 0.03 ^a^	10.58 ± 0.54 ^hij^

Numerical values represent mean ± SEM (standard error of the mean) of three independent experiments. Means followed by different letter(s) within a column represent significantly different values at *p* < 0.05, as determined by Duncan’s new multiple range test

^I, III, IV^ Estimated as halo-zone diameter (mm)/colony diameter (mm)

^II^ Bacterial strains were classified based on SSI as low (SSI < 2.00), intermediate (2.00 < SSI < 4.00), and high (SSI > 4.00) silicate-solubilizers

“-” indicates that the strains did not produce siderophore

### Isolated strains are efficient at silicate and phosphate solubilization

The results of in vitro assays (Table 1) revealed that all 13 strains exhibited beneficial traits that could contribute to plant growth-promotion. Silicate and phosphate solubilization were evaluated based on halo-zone formation on silicate medium (SM) and Pikovskaya’s (PVK) medium, respectively. Figure 1 illustrates representative halo-zone formation by the two candidate strains, NRBC and NRBA, in comparison with the uninoculated control. No halo zone was observed in the uninoculated control (Fig. 1A and B), whereas the bacterial strains produced distinct halo zones on SM (Fig. 1D and G) and PVK medium (Fig. 1E and H), confirming the ability to solubilize inorganic silicate and phosphate, respectively. The highest silicate-solubilizing index (SSI: 4.10) was recorded for the *Acinetobacter baumannii* WRAB isolate. The SSI values for the remaining 12 strains ranged from 2.03 to 3.66. The phosphate-solubilizing index (PSI), on the other hand, ranged from 1.37 to 3.00. Interestingly, the WRAB strain that showed the highest SSI also had significantly elevated PSI. In addition, the NRBC strain also showed significantly elevated PSI values.

The silicate-solubilization potential of two candidate isolates, NRBC and NRBA, was quantified using a broth assay (Table S4). Culture media amended with SiO_2_ (393 mg Si g^− 1^) and field soil (31.5 mg Si g^− 1^) were inoculated with bacterial suspensions and incubated for 7 days. Both bacterial strains significantly increased the soluble silicon content of the culture medium compared with the non-inoculated controls, indicating enhanced solubilization of insoluble silicon sources. In the SiO_2_-amended medium, NRBA and NRBC increased soluble silicon concentrations by 21.74% (97.16 mg Si L^− 1^) and 24.58% (99.43 mg Si L^− 1^), respectively, compared to the control (79.81 mg Si L^− 1^). Although the total silicon content of the field soil was substantially lower than that of SiO_2_, co-cultivation with NRBA and NRBC increased soluble silicon release by 100% (1.60 mg Si L^− 1^) and 48.75% (1.19 mg Si L^− 1^), respectively, relative to the non-inoculated control (0.80 mg Si L^− 1^).

The siderophore production assay showed that seven strains were capable of producing siderophores, as indicated by the color change of Chrome Azurol S (CAS) agar from blue to orange surrounding the colonies. Figure 1C shows the uninoculated control, in which no color change was observed, whereas siderophore production by the candidate strains NRBC and NRBA is shown in Fig. 1F and I. The siderophore production index (SPI) values ranged from 1.24 to 2.23. The seven siderophore-producing strains include all five strains of *Pseudomonas* and two strains of *Burkholderia*. Among them, *B. contaminans* NRBC and *P. aeruginosa* LCPA produced significantly higher amounts of siderophore with SPI values of 2.23 and 2.22, respectively.

All the tested strains were able to produce IAA, ranging from 6.50 to 48.72 µg/mL, when grown in the liquid NB medium amended with 0.15% tryptophan. All *Pseudomonas* strains, except LCPA, produced comparatively higher amounts of IAA compared to other genera. Notably, *P. mosselii* WRPM produced the highest amount of IAA, recording 48.72 µg/mL. Besides, *Burkholderia* sp. NRBA also produced a substantial amount of IAA, with a 24.41 µg/mL concentration. In contrast, *S. surfactantfaciens* WRSS synthesized the least amount of IAA at 6.50 µg/mL.

### *Burkholderia* spp. and *Pseudomonas* spp. possess a higher antifungal property

**Fig. 2:**
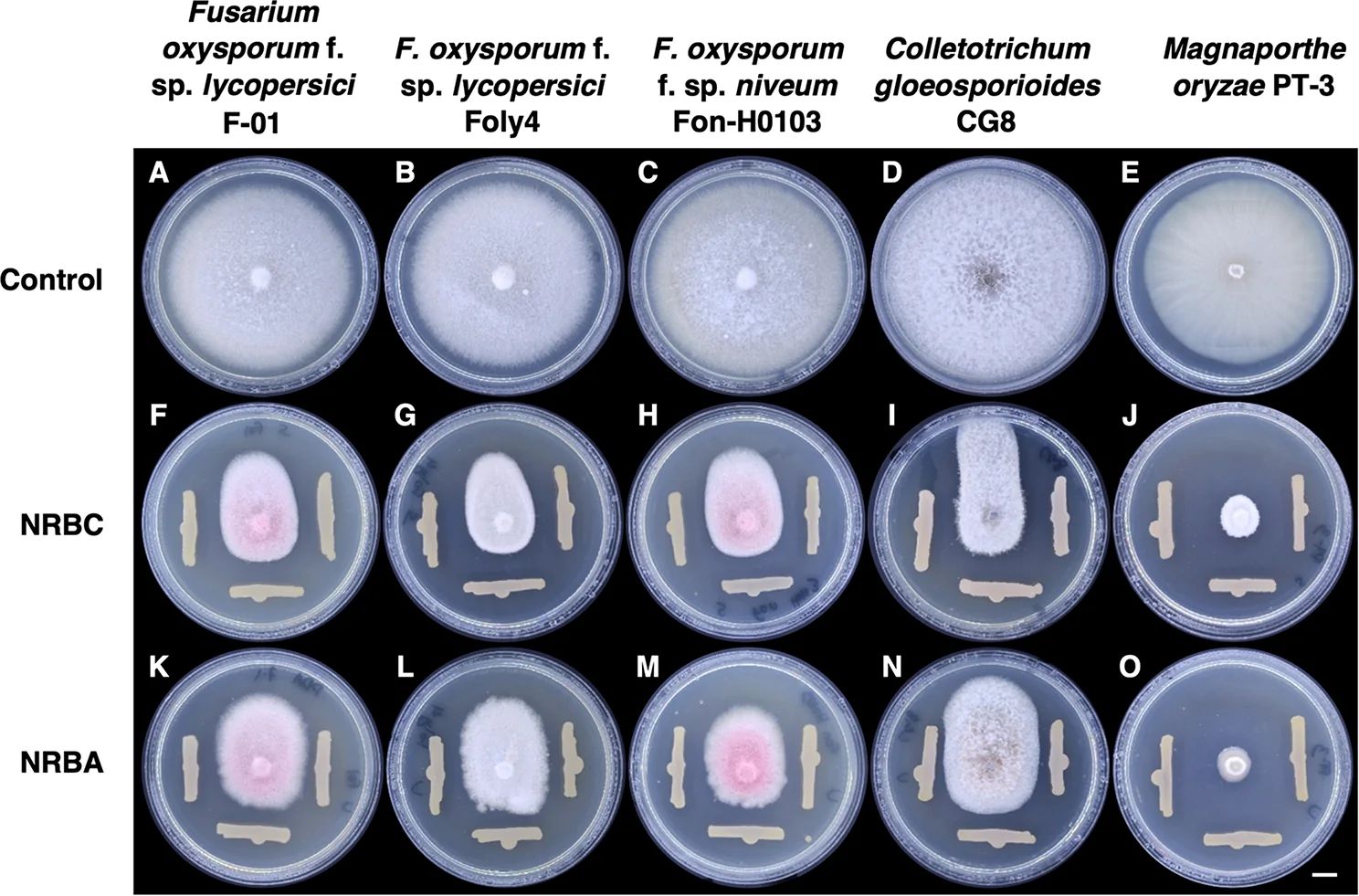
Mycelia growth inhibition of phytopathogens by *Burkholderia contaminans* NRBC and *Burkholderia* sp. NRBA. Bacterial suspensions were streaked on three sides of the potato dextrose agar plates. Water served as control. The fungal disk obtained from an actively growing 10-day-old culture of phytopathogenic fungi was placed at the center of the plate and incubated until the mycelium grew all over the medium on the control plate. The fungal growth inhibition (%) was calculated using the equation ((Rc-R)/Rc) ×100, where “Rc” is the radius of the fungus on the control plate, and “R” is the radius of the fungus on the bacteria-inoculated plate. The experiment consisted of three replicates per trial, and the entire experiment was repeated three times. A representative image from one replication is shown here. A scale bar at the bottom right corner represents 1 cm

The dual culture of bacteria and phytopathogenic fungi on a PDA medium (Fig. 2) showed that *Fusarium oxysporum* f. sp. *lycopersici* Foly4, *F. oxysporum* f. sp. *lycopersici* F-01, *F. oxysporum* f. sp. *niveum* Fon-H0103, and *Colletotrichum gloeosporioides* CG8 were inhibited by the two *Burkholderia* strains (NRBC and NRBA) and one *Pseudomonas* strain (LCPA) (Table 2). Between the two *Burkholderia* strains, NRBC exhibited significantly higher inhibition of Foly4, F-01, Fon-H0103, and CG8. Strain LCPA showed similar inhibition as NRBC against F-01 and CG8 by 67.22% and 66.39%, respectively. These three strains also inhibited the mycelial growth of *Magnaporthe oryzae* PT-3 by more than 82.50%, which indicated their comparatively higher effectiveness against rice blast pathogens. Moreover, PT-3 was inhibited by an additional five strains. Hence, eight out of 13 isolated strains antagonized at least one of the five tested phytopathogens. Notably, all five *Pseudomonas* strains isolated in this study antagonized PT-3. The highest inhibition against PT-3 was demonstrated by two *Pseudomonas* strains, WRPT and LCPA, which inhibited fungal growth by 88.99% and 87.57%, respectively. The least growth inhibition on PT-3 was shown by *Serratia surfactantfaciens* WRSS, with an inhibition of 23.46%.

**Table 2:** In vitro fungal growth inhibition by bacterial strains in dual culture

Bacterial strains	Fungal growth inhibition (%) ^I^				
	*Fusarium oxysporum* f. sp. *lycopersici* Foly4	*Fusarium oxysporum* f. sp. *lycopersici* F-01	*Fusarium oxysporum* f. sp. *niveum* Fon-H0103	*Colletotrichum gloeosporioides* CG8	*Magnaporthe oryzae* PT-3
*Pseudomonas taiwanensis* WRPT	-	-	-	-	88.99 ± 0.38 ^a^
*Serratia surfactantfaciens* WRSS	-	-	-	-	23.46 ± 1.04 ^f^
*P. nitritireducens* WRPN-1	-	-	-	-	44.42 ± 1.37 ^e^
*P. mosselii* WRPM	-	-	-	-	79.09 ± 0.56 ^c^
*P. nitroreducens* WSPN-21	-	-	-	-	51.71 ± 1.03 ^d^
*Burkholderia contaminans* NRBC	63.61 ± 1.67 ^aC^	64.17 ± 0.83 ^aC^	61.67 ± 0.72 ^aC^	67.50 ± 1.38 ^aB^	83.58 ± 0.82 ^bA^
*Burkholderia* sp. NRBA	51.94 ± 4.22 ^bB^	47.50 ± 2.32 ^bB^	51.94 ± 2.76 ^bB^	50.28 ± 0.97 ^bB^	82.50 ± 0.59 ^bA^
*P. aeruginosa* LCPA	52.22 ± 3.90 ^bC^	67.22 ± 0.77 ^aB^	54.17 ± 2.24 ^bC^	66.39 ± 1.11 ^aB^	87.57 ± 0.49 ^aA^

$$^{I}{\rm{Fungal}}\,{\rm{growth}}\,{\rm{inhibition}}\,\left( \%  \right)\, = \,{{{\rm{Radius}}\,{\rm{of}}\,{\rm{the}}\,{\rm{fungal}}\,{\rm{colony}}\,{\rm{in}}\,{\rm{control}} - {\rm{radius}}\,{\rm{of}}\,{\rm{the}}\,{\rm{fungal}}\,{\rm{colony}}\,{\rm{in}}\,{\rm{treatment}}} \over {{\rm{Radius}}\,{\rm{of}}\,{\rm{the}}\,{\rm{fungal}}\,{\rm{colony}}\,{\rm{in}}\,{\rm{control}}}}\, \times \,100{\rm{ }}$$

Values represent mean ± SEM (standard error of the mean). The entire experiment was repeated three times. Means followed by different lowercase letter(s) within a column and different uppercase letter(s) within a row represent significantly different values at *p* < 0.05, as determined by Duncan’s new multiple range test. “-” indicates the inability of strains to inhibit phytopathogens

### MLSA-based phylogenetic characterization of selected isolates

Among the 13 isolated strains, three strains (NRBC, NRBA, and LCPA) exhibited multiple beneficial in vitro traits, including silicate solubilization, phosphate solubilization, siderophore production, IAA production, and antagonistic activity against all tested phytopathogens (Table 1 and Table 2). Among the three strains, NRBC and NRBA were selected for the greenhouse experiments, whereas LCPA was excluded due to its high sequence similarity (100%) to the well-known opportunistic human pathogen *Pseudomonas aeruginosa*.

**Fig. 3:**
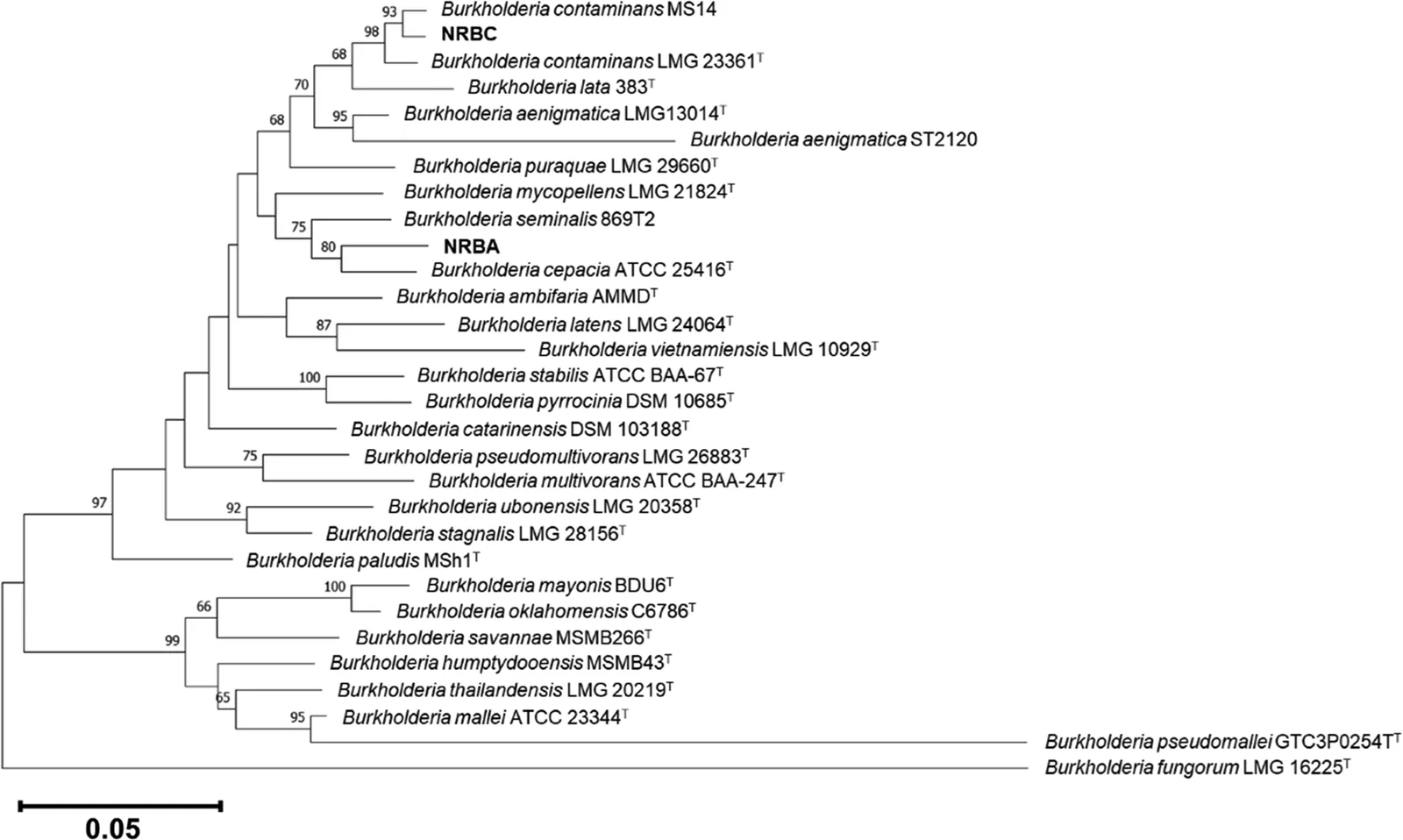
Maximum-likelihood phylogenetic tree based on concatenated sequences (2760 bp) of seven housekeeping genes (*atpD*,* gltB*,* gyrB*,* lepA*,* phaC*,* recA*, and *trpB*) from *Burkholderia* isolates NRBA and NRBC and representative *Burkholderia* species. The tree was reconstructed using the General Time Reversible (GTR) model. Bootstrap values based on 1000 replicates are shown at the nodes when greater than 60%. A discrete Gamma distribution was used to model evolutionary rate differences among sites (5 categories; +G, parameter = 0.2567), and a proportion of sites was assumed to be evolutionarily invariable (+ I, 31.67%). *Burkholderia fungorum* LMG 16225^T^ was used as the outgroup. Scale bar, number of substitutions per site

Based on 16S rRNA gene sequence analysis (Table S1), NRBC and NRBA showed the highest identities to *Burkholderia contaminans* (99.93%) and *B. aenigmatica* (99.71%), respectively. Therefore, a multilocus sequence analysis (MLSA) was used to further reliably differentiate NRBC and NRBA from other closely related *Burkholderia* species. The resulting phylogenetic analysis of concatenated allele sequences of seven housekeeping genes (Fig. 3) revealed that strain NRBC clustered with the type strain *Burkholderia contaminans* LMG 23361^T^ with strong bootstrap value of 98 and shared 99% identity with *B. contaminans* MS14, confirming its taxonomic placement within this species. However, strain NRBA did not cluster with *Burkholderia aenigmatica* as inferred from the 16S rRNA gene analysis. Instead, NRBA grouped with *Burkholderia cepacia* ATCC 25416^T^ with a bootstrap value of 80 and 97% sequence similarity. Due to a 3% divergence of concatenated allele sequence of NRBA from closely related species, it was conservatively designated as *Burkholderia* sp. NRBA.

### *Burkholderia* spp. have the potential to promote the growth of tomato plants and suppress FOL infection under greenhouse conditions

Plant morphology analysis (Table 3) of greenhouse-cultivated tomato plants showed that NRBC + Si treatment significantly increased the shoot dry weight by 22.86% compared to Control + Si treatment. Other growth parameters (plant height, number of leaves, shoot fresh weight, root fresh weight, and root dry weight) were slightly but not significantly increased by the bacterial treatments. On the other hand, under FOL stress (21 dppi), plants treated with bacteria were significantly taller by approximately 10 cm compared to the water treatment (Table 4). No significant improvement was observed for other morphological parameters.

**Table 3:** Effect of bacterial treatments and silicon supplementation on tomato plant growth parameters 21 days after *Fusarium oxysporum* f. sp. *lycopersici* race 2 mock-inoculation

Treatment ^I^	Plant height (cm)	Number of leaves	Shoot		Root	
			Fresh weight (g)	Dry weight (g)	Fresh weight (g)	Dry weight (g)
Control	61.43 ± 2.28 ^a^	8.33 ± 0.42 ^a^	24.91 ± 1.03 ^a^	2.67 ± 0.14 ^ab^	2.24 ± 0.24 ^a^	0.32 ± 0.02 ^a^
Control + Si	61.19 ± 1.46 ^a^	8.90 ± 0.38 ^a^	25.28 ± 1.15 ^a^	2.58 ± 0.14 ^b^	2.25 ± 0.26 ^a^	0.33 ± 0.02 ^a^
NRBC	65.90 ± 1.75 ^a^	9.14 ± 0.32 ^a^	27.46 ± 0.52 ^a^	2.89 ± 0.13 ^ab^	2.71 ± 0.26 ^a^	0.40 ± 0.02 ^a^
NRBC + Si	69.00 ± 2.34 ^a^	9.57 ± 0.36 ^a^	28.09 ± 0.57 ^a^	3.17 ± 0.14 ^a^	2.35 ± 0.31 ^a^	0.38 ± 0.02 ^a^
NRBA	66.62 ± 2.07 ^a^	8.57 ± 0.42 ^a^	25.07 ± 0.98 ^a^	2.61 ± 0.13 ^ab^	2.51 ± 0.30 ^a^	0.33 ± 0.02 ^a^
NRBA + Si	65.62 ± 2.21 ^a^	9.10 ± 0.44 ^a^	25.93 ± 1.05 ^a^	2.71 ± 0.13 ^ab^	2.39 ± 0.28 ^a^	0.35 ± 0.01 ^a^

Values represent mean ± SEM (standard error of the mean). The experiment was conducted with a minimum of five biological replicates (*n* ≥ 5) per trial, and the entire experiment was repeated four times. Means followed by the same letter(s) within a column are not significantly different at *p* < 0.05, as determined by the Kruskal-Wallis test, followed by Dunn’s post-hoc test

^I^ NRBC: *Burkholderia contaminans* NRBC; NRBA: *Burkholderia* sp. NRBA; Si: 2 mM

**Table 4:** Effect of bacterial treatments and silicon supplementation on tomato plant growth parameters 21 days after *Fusarium oxysporum* f. sp. *lycopersici* race 2 (FOL) inoculation

Treatment ^I^	Plant height (cm)	Number of leaves	Shoot		Root	
			Fresh weight (g)	Dry weight (g)	Fresh weight (g)	Dry weight (g)
FOL	54.19 ± 2.75 ^bc^	8.48 ± 0.36 ^a^	22.39 ± 1.03 ^a^	2.12 ± 0.11 ^a^	2.35 ± 0.28 ^a^	0.33 ± 0.01 ^a^
Si + FOL	53.57 ± 2.05 ^c^	8.81 ± 0.36 ^a^	22.76 ± 1.19 ^a^	2.16 ± 0.12 ^a^	2.27 ± 0.31 ^a^	0.30 ± 0.03 ^a^
NRBC + FOL	64.29 ± 2.28 ^a^	10.00 ± 0.53 ^a^	25.55 ± 0.77 ^a^	2.41 ± 0.13 ^a^	2.50 ± 0.28 ^a^	0.34 ± 0.03 ^a^
NRBC + Si + FOL	63.52 ± 2.59 ^a^	9.05 ± 0.42 ^a^	25.01 ± 0.66 ^a^	2.47 ± 0.12 ^a^	2.55 ± 0.19 ^a^	0.34 ± 0.03 ^a^
NRBA + FOL	63.05 ± 2.89 ^ab^	8.52 ± 0.33 ^a^	24.35 ± 1.10 ^a^	2.43 ± 0.14 ^a^	2.50 ± 0.30 ^a^	0.34 ± 0.02 ^a^
NRBA + Si + FOL	63.14 ± 2.04 ^a^	9.62 ± 0.41 ^a^	23.80 ± 0.87 ^a^	2.28 ± 0.11 ^a^	2.49 ± 0.26 ^a^	0.34 ± 0.02 ^a^

Values represent mean ± SEM (standard error of the mean). The experiment was conducted with a minimum of five biological replicates (*n* ≥ 5) per trial, and the entire experiment was repeated four times. Means followed by the same letter(s) within a column are not significantly different at *p* < 0.05, as determined by the Kruskal-Wallis test, followed by Dunn’s post-hoc test

^I^ NRBC: *Burkholderia contaminans* NRBC; NRBA: *Burkholderia* sp. NRBA; Si: 2 mM

**Fig. 4:**
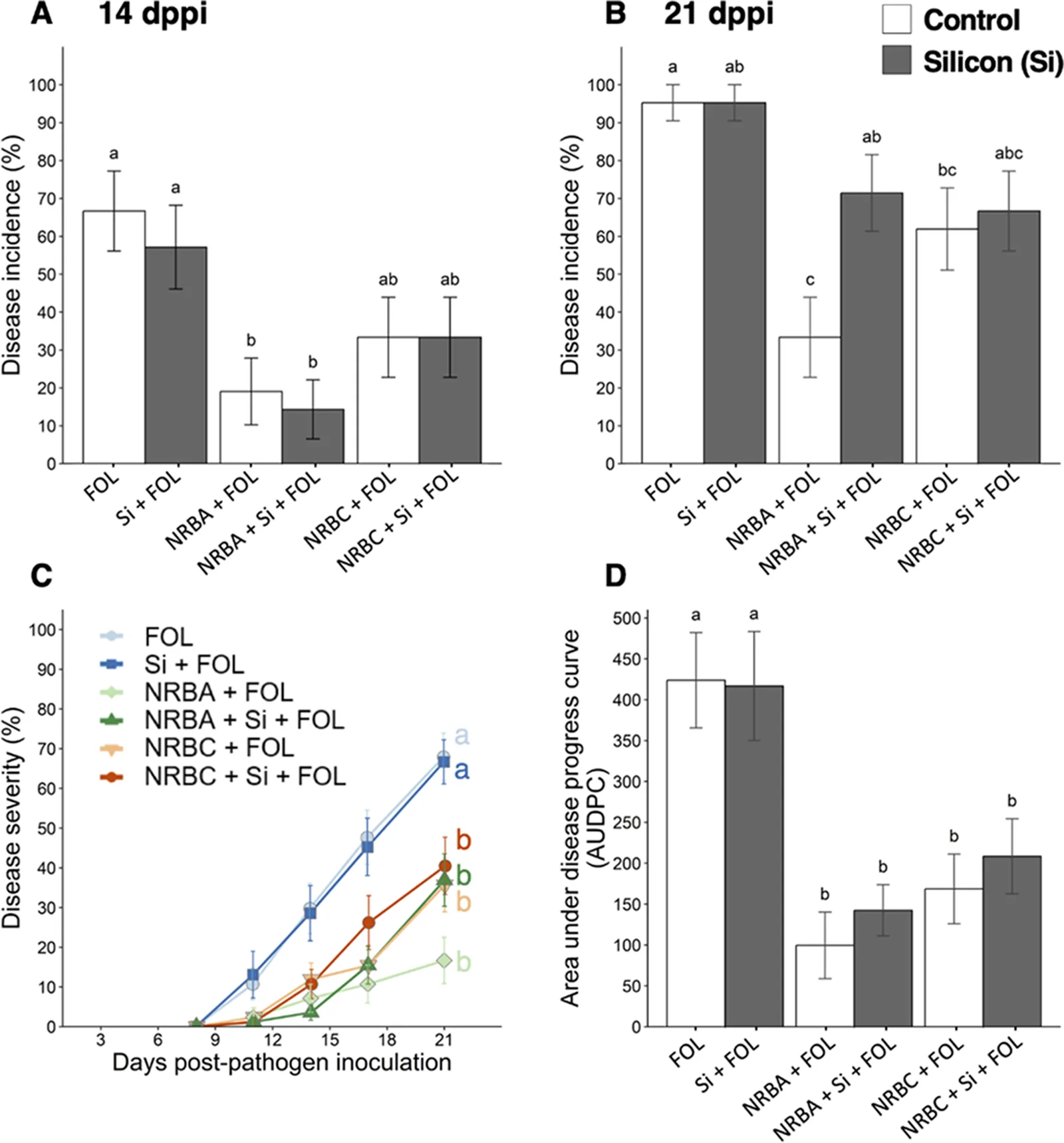
Effects of *Burkholderia* sp. NRBA and *B. contaminans* NRBC, applied with or without silicon (Si), on Fusarium wilt development in tomato under greenhouse conditions. Disease incidence was assessed at (**A**) 14 days post-pathogen inoculation (dppi) and (**B**) 21 dppi. (**C**) Disease severity progression over time. (**D**) Area under the disease progress curve (AUDPC) calculated using the trapezoidal integration method. Data in panels A to C represent the mean ± SEM (standard error of the mean) of 21 biological replicates (*n* = 21) obtained from four independent greenhouse trials. For panel D, AUDPC was calculated separately for each trial using mean disease severity values, and bars represent the mean ± SEM of four independent trials (*n* = 4). Different letters indicate significant differences among treatments (*p* < 0.05) according to the Kruskal-Wallis test followed by Dunn’s post-hoc multiple pairwise comparisons. FOL = *Fusarium oxysporum* f. sp. *lycopersici* race 2

Figure 4A shows that NRBA treatment significantly (*p* < 0.05) prevented the tomato plants from FOL infection at 14 dppi. Although NRBC treatment reduced the wilt incidence by more than 41.68% compared to the control, no significant difference was observed. Notably, NRBA and NRBC alone significantly reduced the disease incidence by 65.02 and 34.98%, respectively, compared to the control at 21 dppi (Fig. 4B). However, the disease incidence under co-application of bacteria and silicon was higher than that of bacteria alone.

The disease progress curve (Fig. 4C) showed that the earliest symptom of Fusarium wilt was observed between 8 and 11 dppi. The symptoms worsened rapidly in the control treatments, resulting in disease severity of 66.7 and 67.9% in Si-supplemented and non-supplemented groups, respectively, at 21 dppi. On the same day, all the bacteria-treated groups exhibited significantly lower disease severity, ranging from 16.7 to 40.5%, compared to the control. Notably, the disease severity was reduced by at least 39% in all bacteria-treated groups compared to the control. The lowest disease severity of 16.7% was recorded under NRBA treatment, with more than 74% reduction compared to the control groups.

The control groups recorded the highest area under disease progress curve (AUDPC) values of 423.81 (without Si supplementation) and 416.67 (under Si supplementation) (Fig. 4D), indicating the higher FOL progression. The bacteria-treated groups resulted in significantly lower AUDPC values than the control, which ranged from 99.40 to 208.33. Among the bacterial treatments, the NRBA treatment had the lowest AUDPC value of 99.40, approximately 76% lower than the control.

The disease suppression ability of NRBA and NRBC was also supported by the reduced FOL recovery from the hypocotyl tissues of bacteria-treated plants (Fig. S2 and Table S5). NRBC treatment exhibited recovery rates of 61.90% and 66.67% in the absence and presence of silicon, respectively. The lowest recovery rate was observed in plants treated with NRBA alone (33.33%), representing a significant reduction compared with the control groups (95.24%). However, the addition of silicon to NRBA increased pathogen recovery to 71.43%. Overall, the re-isolation results demonstrated that both bacterial strains, particularly NRBA, reduced the frequency of FOL recovery from infected plant tissues.

### Expression profiling revealed time-dependent and treatment-specific regulation of defense-related genes under FOL infection

**Fig. 5:**
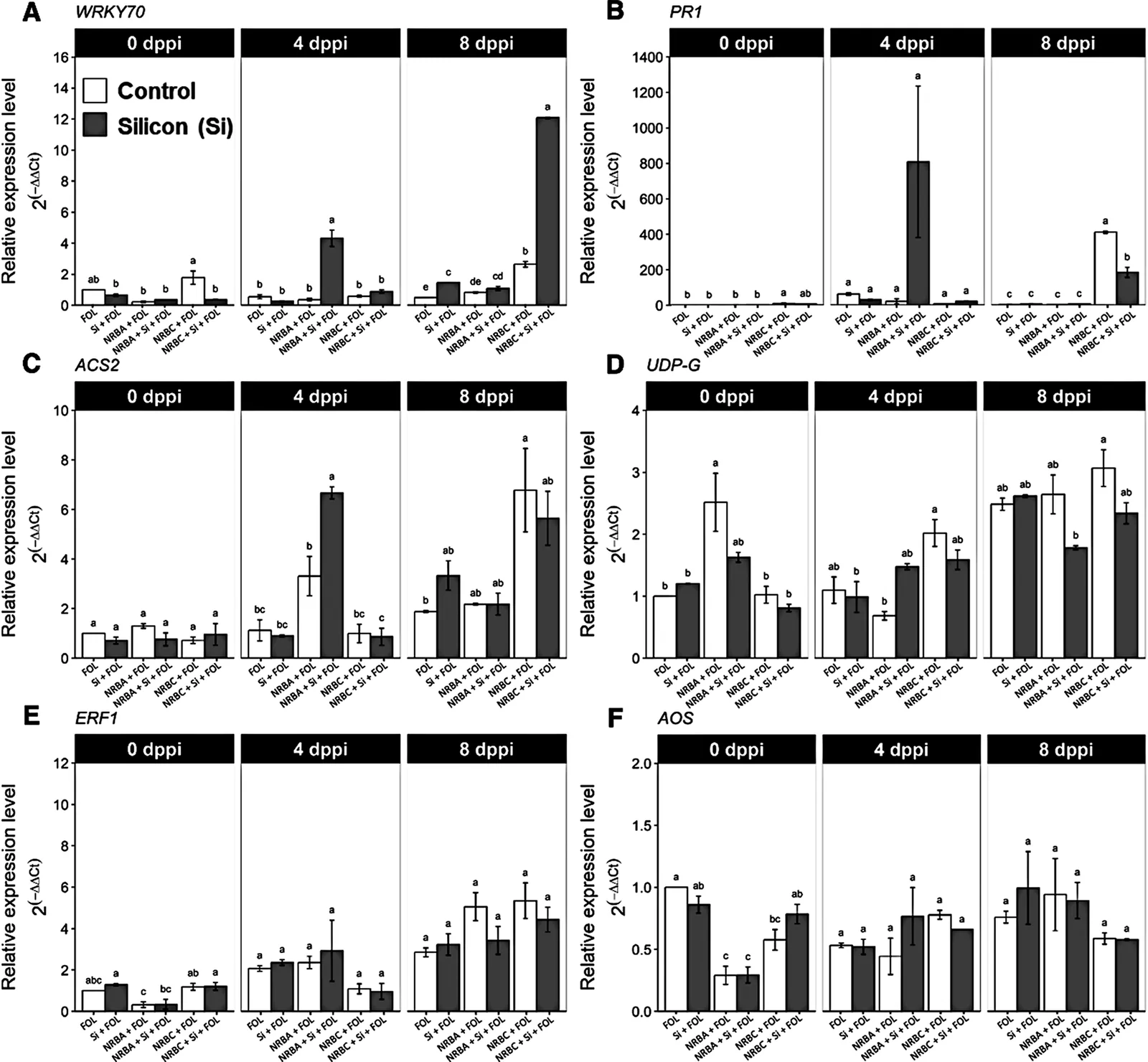
The expression of defense-related genes, including (**A**) *WRKY transcription factor 70* (*WRKY70*), (**B**) *Pathogenesis-related protein 1* (*PR1*), (**C**) *1-aminocyclopropane-1-carboxylate synthase 2* (*ACS2*), (**D**) *Uridine diphosphate-glucosyltransferase* (*UDP-G*), (**E**) *Ethylene Response Factor 1* (*ERF1*), and (**F**) *Allene oxide synthase* (*AOS*) in *Fusarium oxysporum* f. sp. *lycopersici* race 2-infected tomato plants treated with *Burkholderia* sp. NRBA, *B. contaminans* NRBC, and silicon determined by qRT-PCR at 0, 4, and 8 dppi. The *β*-tubulin gene (*TUB*) served as an internal control to normalize the expression level. Values represent mean ± SEM (*n* = 3 biological replicates). Different letters over the error bar represent significantly different values at *p* < 0.05, as determined by Tukey’s honestly significant difference test

At 4 dppi, *WRKY70* (Fig. 5A) and *PR1* (Fig. 5B) were strongly upregulated in the FOL-infected plants that were treated with NRBA + Si, suggesting early involvement of SA-related defense responses. At 8 dppi, however, NRBC-treated groups exhibited higher expression of these genes. The *ACS2* gene (Fig. 5C), a marker of ethylene signaling, showed notable upregulation under NRBA and NRBC treatments at 4 and 8 dppi, respectively. Transcripts of *UDP-G* gene (Fig. 5D), associated with arbutin synthase and antimicrobial responses, increased gradually as the FOL infection progressed to 8 dppi. However, *UDP-G* gene expression was lower in FOL-infected plants under co-treatment of NRBA or NRBC with Si at 8 dppi. The expression of *ERF1* (Fig. 5E) and *AOS* (Fig. 5F), both commonly used as markers of JA/ET-related defense responses, displayed relatively moderate changes throughout the experiment. Collectively, these results suggest that soil inoculation with NRBA and NRBC influences the expression of genes linked to SA and JA/ET-related defense pathways during the tomato response to Fusarium wilt.

## Discussion

Fusarium wilt of tomato, caused by *Fusarium oxysporum* f. sp. *lycopersici* (FOL), is a threat to tomato production (Hernandez-Aparicio et al. 2021; Nabi et al. 2021). Using agrochemicals to control FOL increases production costs, environmental pollution, and associated health risks (Okungbowa and Shittu 2012; Du et al. 2022). Alternatively, the use of beneficial microbes as a biological control agent is a promising approach to managing FOL. In addition, such microbes also enhance nutrient availability, regulate plant physiology, and promote plant growth (O’Rourke et al. 2020; Heo et al. 2022; Jia et al. 2022; Pazarlar et al. 2022).

In this study, 13 rhizobacterial strains with the potential to be used as alternatives to fungicides in managing plant stresses were obtained. Most of the bacterial strains possessed multiple plant growth-promoting properties (Fig. 1 and Table 1) such as silicate solubilization, phosphate solubilization, IAA production, siderophore production, and inhibition of phytopathogens (Fig. 2 and Table 2). Most of the bacterial strains were isolated from the rice rhizosphere. Since rice is a high silicon accumulator, its rhizosphere is an ideal habitat for silicate-solubilizing bacteria (SSB) (Chandrakala et al. 2019). All the strains isolated from the rice rhizosphere demonstrated intermediate and high silicate-solubilizing indices (SSI), indicating their efficiency in solubilizing silicon. Hence, the rice plants and SSB have synergistic interactions. Interestingly, all the SSB obtained in our study could also solubilize phosphate. Previously, *Pseudomonas*,* Bacillus*,* Burkholderia*, and *Enterobacter* have been reported to possess dual (silicate and phosphate solubilizing) properties (Kang et al. 2017; Chandrakala et al. 2019; Lee et al. 2019; Adhikari et al. 2020; Bist et al. 2020; Cruz et al. 2022), but none of the *Burkholderia* strains isolated in this study has been previously reported for their silicate solubilizing abilities.

Among the isolated stains, NRBA, NRBC, and LCPA exhibited broad-spectrum antagonistic activity against all tested phytopathogens and were therefore considered promising candidates for greenhouse evaluation. However, LCPA was excluded from further studies because 16S rRNA gene sequence analysis revealed a high similarity (100%) to *P. aeruginosa* (Table S2), a well-known opportunistic human pathogen (Reyne et al. 2023). In contrast, NRBA and NRBC were classified within the *Burkholderia cepacia* complex (Bcc), showing the highest 16S rRNA gene sequence similarity to *Burkholderia aenigmatica* (99.71%) and *B. contaminans* (99.93%), respectively. The Bcc comprises a group of closely related gram-negative bacteria that have attracted considerable attention for their roles in biological control, bioremediation, and plant growth promotion. However, their biotechnological application has been limited due to the opportunistic pathogenic nature of some Bcc members (Coenye and Vandamme 2003; Mahenthiralingam et al. 2005). Numerous Bcc members, including *B. contaminans* MS14 (Deng et al. 2017), *B.* *lata* 383 (Vanlaere et al. 2009), *B.* *ambifaria* AMMD (Coenye and Vandamme 2003), *B. seminalis* (Hwang et al. 2021), and *B. puraquae* sp. nov. LMG 29660 (Martina et al. 2018), have been reported to confer agricultural benefits. In contrast, *Burkholderia* species such as *B. mallei*, *B. pseudomallei*, *B. multivorans*, *B. oklahomensis*, and *B. cenocepacia* include strains associated with opportunistic infections (Coenye and Vandamme 2003; Deng et al. 2016). Taxonomic assignment within the Bcc is challenging because closely related species often share highly similar 16S rRNA gene sequences. Therefore, multilocus sequence analysis (MLSA) based on seven housekeeping genes was performed to achieve higher phylogenetic resolution (Fig. 3). MLSA confirmed that NRBC clustered closely with *B. contaminans* and showed high similarity to strain MS14, which has been reported to possess biocontrol activity (Deng et al. 2016, 2017). In contrast, NRBA grouped within the *B. cepacia* lineage but was supported by a moderate bootstrap value (80%) and shared 97% MLSA sequence identity with the *B. cepacia* lineage, suggesting that its precise taxonomic position remains unresolved. Since a 3% divergence among the concatenated allele sequences corresponds to the proposed species delineation threshold for Bcc members (Vanlaere et al. 2009), NRBA was conservatively designated as *Burkholderia* sp. NRBA. Given the substantial genetic diversity within *Burkholderia* and the presence of both beneficial and opportunistic pathogenic members, accurate species-level identification using a combination of MLSA, whole-genome sequencing, average nucleotide identity (ANI), and DNA-DNA hybridization analyses, and biosafety assessments are essential when evaluating strains for agricultural applications. Furthermore, comparative genomic analyses of the isolated strains alongside representative *Burkholderia* genomes can facilitate the identification of homologous gene clusters associated with several functions, such as human virulence (VIR) and environmental adaptation and plant-microbe interactions (ENV). Hung et al. (2026) successfully applied this approach to reclassify *Burkholderia seminalis* strain 869T2 into a safer lineage, thereby resolving the biosafety concerns associated with its initial classification as *Burkholderia cepacia*. Hence, comparative genomic analysis can facilitate the discrimination of potentially opportunistic pathogens from environmentally adapted strains with greater potential for safe agricultural applications.

The two most promising *Burkholderia* strains (NRBC and NRBA) based on in vitro beneficial traits were selected for greenhouse experiments. Under normal conditions without FOL inoculation, the NRBC strain resulted in significantly higher shoot dry biomass under 2 mM Si supplementation compared to the non-supplemented control. These findings align with Zhang et al. (2018), who reported that 2.5 mM silicon improved the biomass of tomato seedlings due to improved photosynthesis rate. Greger et al. (2018) found that Si could increase the P availability in the soil by 50%. Since NRBC also demonstrated phosphate-solubilizing ability, its co-application with Si might have enhanced plant-available phosphorus in the rhizosphere, resulting in increased shoot dry weight. Hence, although tomato is a poor silicon accumulator, it can benefit from Si supplementation through an indirect mechanism, regulating macronutrients and micronutrients in the soil.

Under FOL stress, the control (water-treated) groups showed stunted growth, whereas the bacteria-treated tomato plants were significantly taller due to effective suppression of FOL (Table 4). Applying the NRBC strain significantly increased shoot dry weight under normal conditions (Table 3), but it could not exhibit higher biomass under FOL stress. In general, plant resource allocation favors growth. However, during a pathogen attack, resources are generally diverted toward defense mechanisms (Monson et al. 2022). Such a trade-off of plant nutrients between growth and defense response is requisite for survival (Ha et al. 2021). Therefore, plant nutrient and defense-related enzyme analyses could elucidate the relation between resource allocation towards growth and defense during disease progression. Results of Fusarium inoculation (Fig. 4) show that both NRBA and NRBC reduce disease incidence and the area under the disease progress curve (AUDPC). However, the addition of silicon lessened the protective ability of NRBA. Reduced FOL recovery from hypocotyl tissues of bacteria-treated plants further supports the disease-suppressive effects of NRBA and NRBC compared to water-treated control (Fig. S2 and Table S5), suggesting that these strains may suppress pathogen colonization through direct antagonism. NRBA exhibited the highest suppression, reducing pathogen recovery by approximately two-thirds compared with the pathogen control. This finding is consistent with the lower disease incidence and severity (Fig. 4) observed in NRBA-treated plants and supports its potential role as an effective biocontrol agent against Fusarium wilt.

The abilities of biocontrol agents to produce IAA and siderophores have suppressive effects on FOL (Ruiz et al. 2015; Simonetti et al. 2018). Similarly, Abbasi et al. (2019) have found a significant correlation between plant growth-promoting traits (IAA and siderophore production) and inhibition of FOL by *Streptomyces* strains. IAA plays a crucial role in the modification of root architecture, vascular differentiation, and disease signaling (Ambrosini and Passaglia 2017). Siderophores enhance the availability of iron (Fe) for plants by sequestering it from the environment, which competitively suppresses phytopathogens (Kumar et al. 2018). In our study, both NRBA and NRBC antagonized all tested phytopathogens, including FOL, in a dual plate assay, indicating the production of antifungal compounds. Therefore, the ability of *Burkholderia* strains (NRBA and NRBC) to produce IAA, siderophores, and antifungal compounds potentially mitigated the FOL stress through nutrient competition and direct antagonism. Hence, future studies may focus on identifying the key antifungal compounds produced by these strains to elucidate the direct antifungal mechanism. In addition, quantification of FOL biomass by qPCR, together with assessments of bacterial colonization and persistence within plant tissues, would provide deeper insights into the mechanism underlying FOL suppression.

In addition to direct antagonism, beneficial microbes may enhance plant resistance against phytopathogens by modulating host defense responses. Therefore, the relative expression of six defense-related genes (*WRKY70*, *PR1*, *ACS2*, *UDP-G*, *ERF1*, and *AOS*) was evaluated in FOL-infected tomato plants (Fig. 5). *WRKY70* and *PR1* are key marker genes of SA-related defense responses, whereas *ACS2*, *ERF1*, and *AOS* are associated with JA/ET signaling. *UDP-G* has been implicated in antimicrobial responses and cell wall-associated defense mechanisms. In this study, the bacteria + Si treatments showed pronounced upregulation of *WRKY70* and *PR1*. However, these same treatments exhibited higher disease incidence and severity than bacteria-only treatments. The contrasting disease outcomes between the bacteria-only and bacteria + Si treatments suggest that silicon co-application may have altered the balance of defense-related signaling pathways. This interpretation is consistent with the expression profiles of *ACS2* (Fig. 5C), *ERF1* (Fig. 5E), and *AOS* (Fig. 5F). Notably, *ERF1* expression at 8 dppi was lower in the bacteria + Si treatments than in the corresponding bacterial treatments (Fig. 5E), suggesting that silicon may have influenced the transcriptional response elicited by bacterial inoculation. Additionally, the altered expression of *UDP-G* (Fig. 5D) suggests that the bacterial treatments may have influenced defense-related metabolic processes, including cell wall reinforcement and pathogen responses (Kuzniak et al. 2015).

Collectively, these findings indicate that the sole application of NRBA or NRBC enhances tomato plants’ defense against FOL and alters the expression of defense-related genes in tomato, whereas their co-application with silicon may be counterproductive under Fusarium wilt stress. Both *Burkholderia* isolates exhibited antifungal activity in vitro and reduced disease progression under greenhouse conditions; however, the current findings do not fully differentiate whether the achieved disease suppression is through direct antifungal activity of the bacterial strains or the host-mediated induced resistance. Therefore, identification of antifungal compounds produced by these strains could elucidate the direct antifungal mechanism. Although changes in the expression of SA- and JA/ET-associated marker genes were observed, the present data do not provide direct *in planta* validation of these signaling pathways. The gene expression analysis was performed using hypocotyl tissues collected at selected post-inoculation time points. Although this sampling strategy captured changes in gene expression over time, it may not fully represent the overall plant defense response during disease development. Future studies incorporating additional tissues, a broader range of sampling time points, and hormone quantification, particularly during the early stages of infection, would improve the understanding of the defense responses and transcriptional changes observed in this study. In addition, quantification of silicon in both plant tissues and soil is needed to clarify the role of silicon in FOL suppression. Furthermore, analyses of defense-related enzymes, including superoxide dismutase (SOD), catalase (CAT), peroxidase (POD), and polyphenol oxidase (PPO), would provide valuable insights into the mechanisms underlying disease suppression.

## Conclusions

In this study, 13 silicate-solubilizing bacteria (SSB) possessing plant growth-promoting (PGP) traits were isolated, characterized, and identified. Two *Burkholderia* strains, NRBC and NRBA, with superior PGP and antifungal traits were selected for greenhouse evaluation and demonstrated effective biocontrol against Fusarium wilt of tomato caused by *F. oxysporum* f. sp. *lycopersici* (FOL) race 2. Contrary to our expectation, silicon supplementation did not provide the anticipated plant growth promotion of tomato plants and FOL suppression. Although bacterial treatments influenced the expression of genes linked to SA and JA/ET-related defense pathways, the underlying mechanisms of disease suppression could not be fully elucidated. Therefore, future studies involving plant hormone, plant and soil nutrient quantification, together with analyses of defense-related enzymes, would clarify the mechanisms involved. Overall, NRBC and NRBA are promising candidates for the biological control of tomato Fusarium wilt. However, since these strains belong to the *Burkholderia cepacia* complex, future studies should incorporate whole-genome sequencing for accurate species-level identification and comprehensive biosafety assessments before their agricultural application.

## Supplementary Information

Below is the link to the electronic supplementary material.


Supplementary Material 1


## Data Availability

All data generated or analyzed during this study are included in this published article.

## Abbreviations

*ACS2*: *1-aminocyclopropane-1-carboxylate synthase 2*
*AOS*: *Allene oxide synthase*
AUDPC: Area under the disease progress curve
BCAs: Biocontrol agents
CFU: Colony-forming unit
CRD: Completely randomized design
DAT: Days after transplanting
dH_2_O: Distilled water
dppi: Days post-pathogen inoculation
*ERF1*: *Ethylene Response Factor 1*
FOL: *Fusarium oxysporum* f. sp. *lycopersici*
*PR1*: *Pathogenesis-related protein 1*
PSI: Phosphate-solubilizing index
SA: Salicylic acid
SPI: Siderophore production index
SSB: Silicate-solubilizing bacteria
SSI: Silicate-solubilizing index
TSB: Tryptic soy broth
*TUB*: *β-tubulin*
*UDP-G*: *UDP-glycosyltransferase*
*WRKY70*: *WRKY transcription factor 70*
