# Supplementary material for: Biocontrol potential of Burkholderia spp. against Fusarium oxysporum f. sp. lycopersici infecting tomato
Source: Bot Stud. 2026 Aug 28;67:10. doi: 10.1186/s40529-026-00497-z (PMC13525146; doi:10.1186/s40529-026-00497-z)
Supplement: Supplementary file 1 [file 40529_2026_497_MOESM1_ESM.docx]

**Table S1.** Details of fungal phytopathogens used in this study

| Host plant | Scientific name | Isolate | Provider |
| --- | --- | --- | --- |
| Tomato | *Fusarium oxysporum* f. sp. *lycopersici* | Foly4 | World vegetable center |
| Tomato | *F. oxysporum* f. sp. *lycopersici* | F-01 | Taiwan seed improvement and propagation station (TSIPS) |
| Watermelon | *F. oxysporum* f. sp. *niveum* | Fon-H0103 | Dr. Jenn-Wen Huang |
| Strawberry | *Colletotrichum gloeosporioides* | CG8 | Dr. Wen-Hsin Chung |
| Rice | *Magnaporthe oryzae* | PT-3 | Dr. Chih-Li Wang |

**Table S2**. Molecular identity of 18 rhizobacterial strains based on sequence analysis of 16S rDNA region

| Strain ^I^ | Closest identity ^II^ | Identity (%) |
| --- | --- | --- |
| LCKV | *Klebsiella variicola* F2R9 | 99.93 |
| LCAP | *Acinetobacter pittii* DSM | 99.86 |
| LCPN-23 | *Pseudomonas nitroreducens* IAM | 99.35 |
| LCPA | *P. aeruginosa* DSM | 100.00 |
| NRAP-1 | *A. pittii* strain LMG | 99.93 |
| NRAP-2 | *A. pittii* strain LMG | 99.93 |
| NRBC | *Burkholderia contaminans* J2956 | 99.93 |
| NRBA | *B. aenigmatica* LMG | 99.71 |
| NRKV | *K. variicola* F2R9 | 99.85 |
| NRRS | *Ralstonia syzygii* subsp. *indonesiensis* UQRS | 99.06 |
| NRAP-3 | *A. pittii* DSM | 99.86 |
| WRPT | *P. taiwanensis* DSM 21245 | 100.00 |
| WRSS | *Serratia surfactantfaciens* YD25 | 99.86 |
| WRPN-1 | *P. nitritireducens* WZBFD3-5A2 | 99.49 |
| WRAB | *A. baumannii* ATCC | 100.00 |
| WRPM | *P. mosselii* CFML | 99.93 |
| WSPN-21 | *P. nitroreducens* IAM | 99.35 |
| WSPN-22 | *P. nitroreducens* IAM | 99.35 |

^I^ The first two alphabets, LC, NR, WR, and WS, of strain represent the source as Linluo cucumber, NPUST rice, Wandan rice, and Wandan sugarcane fields, respectively. The last two alphabets of strain are the initials of their scientific names.

^II^ The closest identity was obtained by aligning the sequence of self-isolated strains to the NCBI BLAST database.

**Table S3.** List of primers used for qRT-PCR

| Gene | Forward Primer (5’ to 3’) | Reverse Primer (3’ to 5’) |
| --- | --- | --- |
| *β*-tubulin (*TUB*) | CAAGAACTCGTCCTACTTTG | GCTCACTCACCCTTCTAA |
| *WRKY transcription factor 70* (*WRKY70*) | TGGTAAAGCATAGTGACTCAAC | AGAGGGAGAAGAAGGCATAA |
| *Pathogenesis-related protein 1* (*PR1*)  *1-aminocyclopropane-1-carboxylate synthase 2* (*ACS2*) | GGTAACTGGAGAGGACAA  GATGGATTTGCGTCCACTTT | GACAATCGATCACTTTATTC  GATCCAGGCGAGACGTTAG |
| *UDP-glycosyltransferase* (*UDP-G*) | GATGAACGCCACCTTCTTAG | CTCCTTCCATAACAATCCTCAC |
| *Ethylene Response Factor 1* (*ERF1*) | AGACTTGGGAGTTGAATTA | TACATTGCGATCTTGATTA |
| *Allene oxide synthase* (*AOS*) | CCTCTTCCTTCTCTTCACCAAA | GCCGGGTATAGTCCTGGTAGA |

**Table S4.** Silicon (Si) content in insoluble sources and solubilized Si content in the culture medium due to dissolution by SSB

| Substrates | Total Si content in the substrate  (mg Si g^-1^) | Total Si added to culture medium  (mg Si L^-1^) | Solubilized Si content in culture medium ^I^ | | |
| --- | --- | --- | --- | --- | --- |
|  |  |  | Control without bacteria inoculation  (mg Si L^-1^) | After incubation with NRBA for 7 days  (mg Si L^-1^) | After incubation with NRBC for 7 days  (mg Si L^-1^) |
| SiO_2_ | 393 | 982.50 | 79.81 ± 0.39 ^b^ | 97.16 ± 0.63 ^a^ | 99.43 ± 0.68 ^a^ |
| Soil | 31.5 | 78.75 | 0.80 ± 0.16 ^b^ | 1.60 ± 0.16 ^a^ | 1.19 ± 0.06 ^ab^ |

^I^ Values represent mean ± SEM (standard error of the mean). The experiment was conducted with three replications and repeated three times. Means followed by different lowercase letter(s) within a row represent significantly different values at *p* < 0.05, as determined by Tukey's honestly significant difference test.

**Table S5.** Recovery of *F. oxysporum* f. sp. *lycopersici* (FOL) on peptone-pentachloronitrobenzene (PCNB) medium from the hypocotyl of FOL-challenged plants, which were co-treated with bacteria (*Burkholderia contaminans* NRBC and *Burkholderia* sp. NRBA) and 2 mM silicon (Si)

| Treatment | Recovery (%) ^I^ |
| --- | --- |
|  |  |
| FOL | 95.24 ± 4.76 ^a^ |
| Si + FOL | 95.24 ± 4.76 ^ab^ |
| NRBA + FOL | 33.33 ± 10.54 ^c^ |
| NRBA + Si + FOL | 71.43 ± 10.10 ^ab^ |
| NRBC + FOL | 61.90 ± 10.86 ^bc^ |
| NRBC + Si + FOL | 66.67 ± 10.54 ^abc^ |

^I^ Recovery (%) = $\frac{Number of hypocotyl exhibiting fungal growth}{Total number of hypocotyl}$×100

Values represent mean ± SEM (standard error of the mean). The experiment was conducted with a minimum of five biological replicates (n ≥ 5) per trial, and the entire experiment was repeated four times. Means followed by the same letter(s) within a column are not significantly different at *p* < 0.05, as determined by the Kruskal-Wallis test, followed by Dunn’s post-hoc test.

NRBC: *Burkholderia contaminans* NRBC; NRBA: *Burkholderia* sp. NRBA; Si: 2 mM.

**Fig. S1.** Map of Taiwan showing the four sampling sites in Pingtung County. Two of the fields were being used for rice cultivation and had been recently harvested, namely, Wandan rice field (GPS coordinate: 22°36'56.3"N 120°29'19.1"E) and NPUST rice field (GPS coordinate: 22°38'38.0"N 120°36'18.2"E). The other two fields were still under cultivation, namely, Linluo cucumber field (GPS coordinate: 22°37'56.2"N 120°32'09.6"E) and Wandan sugarcane field (GPS coordinate: 22°36'57.2"N 120°29'17.2"E).

**Fig. S2.** Recovery of *Fusarium oxysporum* f. sp. *lycopersici* (FOL) from the FOL-challenged plants on peptone-pentachloronitrobenzene (PCNB) medium.

The treatments included control groups treated with water (A, D), bacterial treated groups inoculated with *Burkholderia contaminans* NRBC (B, E), and *Burkholderia* sp*.* NRBA (C, F). Additionally, the plants were subjected to either 2 mM silicon (Si) (D, E, F) or a mock fertilizer treatment (Control) (A, B, C).
